# Model of the Dynamic Construction Process of Texts and Scaling Laws of Words Organization in Language Systems

**DOI:** 10.1371/journal.pone.0168971

**Published:** 2016-12-22

**Authors:** Shan Li, Ruokuang Lin, Chunhua Bian, Qianli D. Y. Ma, Plamen Ch. Ivanov

**Affiliations:** 1 Institute for Biomedical Electronics Engineering, School of Electronic Science and Engineering, Ministry of Education Key Laboratory of Modern Acoustics, Nanjing University, Nanjing 210093, China; 2 Keck Laboratory for Network Physiology, Department of Physics, Boston University, Boston, MA 02215, United States of America; 3 Department of Neurology and Program in Neuroscience, Harvard Medical School, Beth Israel Deaconess Medical Center, Boston, MA 02215, United States of America; 4 College of Geographic and Biologic Information, Nanjing University of Posts and Telecommunications, Nanjing 210003, China; 5 Harvard Medical School and Division of Sleep Medicine, Brigham and Women’s Hospital, Boston, MA 02115, United States of America; 6 Institute of Solid State Physics, Bulgarian Academy of Sciences, Sofia 1784, Bulgaria; University of Rijeka, CROATIA

## Abstract

Scaling laws characterize diverse complex systems in a broad range of fields, including physics, biology, finance, and social science. The human language is another example of a complex system of words organization. Studies on written texts have shown that scaling laws characterize the occurrence frequency of words, words rank, and the growth of distinct words with increasing text length. However, these studies have mainly concentrated on the western linguistic systems, and the laws that govern the lexical organization, structure and dynamics of the Chinese language remain not well understood. Here we study a database of Chinese and English language books. We report that three distinct scaling laws characterize words organization in the Chinese language. We find that these scaling laws have different exponents and crossover behaviors compared to English texts, indicating different words organization and dynamics of words in the process of text growth. We propose a stochastic feedback model of words organization and text growth, which successfully accounts for the empirically observed scaling laws with their corresponding scaling exponents and characteristic crossover regimes. Further, by varying key model parameters, we reproduce differences in the organization and scaling laws of words between the Chinese and English language. We also identify functional relationships between model parameters and the empirically observed scaling exponents, thus providing new insights into the words organization and growth dynamics in the Chinese and English language.

## Introduction

Scaling laws have been discovered and investigated in many fields such as physics, biology, finance, geology, and sociology. Examples include urban growth [1], population distribution of cities [2, 3], clusters in DNA structure [4, 5], financial markets [6–8], spatial distribution of words in texts [9–13], structures of rocks and geological formations [14], citation networks [15], social interactions [16], and stochastic physical systems [17, 18]. Zipf’s and Heaps’ law, two classical representatives of scaling laws, have been studied in various systems [19–22]. In the context of natural language structure and organization, Zipf’s law indicates the inversely proportional relationship in log-log scale between the descending words frequency *Z*(*r*) and the words frequency rank *r* [23]. Heaps’ law reveals a different aspect of words organization in natural languages, which indicates that the vocabulary size *N*(*t*) of a given text grows roughly as a sub-linear function in log-log plot with increasing text length *t* [24]. The initial Zipf’s study focused on the English language [23], and later researchers extended his work to other natural languages, including Hebrew, Greek, Spanish and Irish [25, 26], reporting that different languages are characterized by distinct scaling exponents, reflecting differences in words organization.

Although Chinese is a widely used language, relatively few studies have focused on the Chinese lexical organization [21, 27], and the laws which govern words frequency, words rank and growth of distinct words with increasing text length in the Chinese language remain not well understood. The existence of Chinese polysemic words and the complex word segmentation rules in the Chinese language pose challenges to systematic and consistent studies [28]. Currently, it is an open question whether Zipf’s law, Heaps’ law or other scaling laws adequately describe the structure and organization of the Chinese language system.

In this study, we analyze scaling properties of words organization in the Chinese language and compare with English, using a database of classic Chinese and English books. Our analyses show that: (i) the probability distribution of words frequency in the Chinese and English language obeys a power-law, and the word organization of both languages conforms to Zipf’s law and Heaps’ law; (ii) the scaling exponents and crossover behavior of these two languages are significantly different, reflecting the different role and organization of Chinese characters compared to English words. Furthermore, we propose a model of words organization and text growth mechanism which accounts for the empirically observed scaling laws. The introduced model parameters provide new insight into the scaling dynamics and construction mechanisms of words organization in the Chinese and English written texts. By varying key model parameters, we successfully reproduce the differences in the organization and scaling properties of words in Chinese and English texts, and we establish functional relationship between the empirically observed scaling exponents and our model parameters.

## Methods and Results

### Database

We compare two categories of data sets obtained from Project Gutenberg [29]:

1. Ten classic Chinese books written by different authors during the period from the 14th to late 18th century, including novels, chinese mythologies and history texts, where each book has approximately 0.4 × 10^4^ distinct Chinese characters and on average 5 × 10^5^ total number of Chinese characters per book (a Chinese character can appear many times in a book). In our analyses, we treat each Chinese character as a separate word because in contrast to western languages where each word is composed of letters, characters in the Chinese language do not correspond to letters but often indicate separate words, and the same Chinese character can play role as a verb, noun, or adverb depending on the context in the sentence.
2. Ten classic English books written by different authors covering different themes and genres. Each book contains approximately 10^4^ distinct words and 10^5^ text length, a corpus of words comparable in size with the database of Chinese classic books analyzed in this paper.

Detailed information on the books included in our analyses, their length and the vocabulary size of each book is shown in Table 1.

**Table 1:** Detailed information on the database of Chinese and English books and corresponding words statistics for each book. *T* is the total number of words, and *N*_*T*_ is the vocabulary size of each book.

Language	No.	Book Title	*T*	*N*_*T*_
Chinese	01	Flowers in the Mirror 镜花缘(by *Ruzhen Li* 李汝珍)	338469	4229
	02	The Scholars 儒林外史(by *Jingzi Wu* 吴敬梓)	231950	3338
	03	Sigong’an 施公案(by *Anonymous* 佚名)	967788	4077
	04	Amazing Tales 二刻拍案惊奇(by *Mengchu Ling* 凌初)	52559	4241
	05	Yuewei Cottage Notes 阅微草堂笔记(by *Xiaolan Ji* 纪晓岚)	307714	4980
	06	Penggong’an 彭公案(by *Tan Meng Dao Ren* 贪梦道人)	732458	3481
	07	Stories to Caution the World 警世通言(by *Menglong Feng* 冯梦龙)	312085	4333
	08	Journey to the West 西游记(by *Chengen Wu* 吴承恩)	589705	4483
	09	Dream of Red Mansions 红楼梦(by *XueQin Cao* 曹雪芹)	730112	4636
	10	Romance of the Three Kingdoms 三国演义(by *Guanzhong Luo* 罗贯中)	485914	3950
English	01	Alices adventures in wonderland (by *Lewis Carroll*)	30083	4285
	02	Ulysses (by *James Joyce*)	268843	29020
	03	Hamlet (by *William Shakespeare*)	218510	16963
	04	David Crockett (by *John S. C. Abbott*)	78802	7343
	05	A Christmas carol (by *Charles Dickens*)	57314	4778
	06	The adventures of Tom Sawyer (by *Mark Twain*)	27341	2572
	07	Moby-Dick; Or, the Whale (by *Herman Melville*)	59909	5546
	08	The hound of the Baskerville (by *Sir Arthur Conan Doyle*)	73908	7172
	09	An enquiry concerning human understanding (by *David Hume*)	30979	4834
	10	The origin of species by means of natural selection (by *Charles Darwin*)	156815	6930

### Fundamental scaling laws of words organization in Chinese language

**Fig 1:**
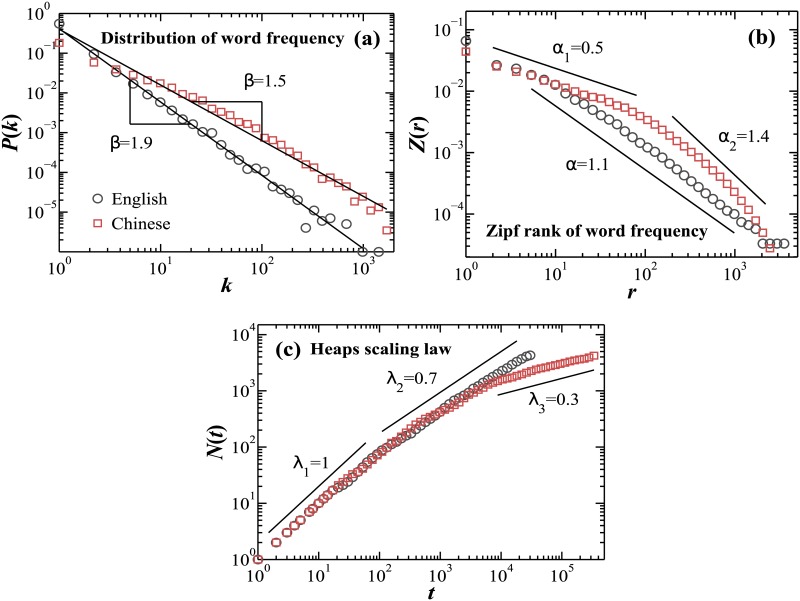
Scaling analyses of words organization in Chinese and English books. Words organization in Chinese and English language exhibits scaling laws with different characteristics. Log-log plots of (a) probability distribution *P*(*k*) of the word frequency *k*, (b) Zipf’s law *Z*(*r*) of the word frequency rank *r*, and (c) Heaps’ scaling law of the number of distinct words *N*(*t*) vs. the number of words *t* in the text, obtained for the first book in Chinese and in English language listed in Table 1. Straight lines indicate the fitting range where the scaling exponents *β*, *α* and λ are obtained. Our analyses show that (a) Chinese language books exhibit lower exponent *β* compared to English books; (b) while English books exhibit a single scaling regime over the entire range of frequency ranks *r*, Chinese texts are characterized by a clear crossover in the Zipf’s scaling of the normalized word frequency *Z*(*r*) vs. word frequency rank *r*; (c) the number of distinct words *N*(*t*) vs. text length *t* exhibits a crossover with two different scaling exponents at small and intermediate scales for both Chinese and English books. However, Chinese texts are characterized by a third saturation regime for large scales *t* that is not observed for English books.

We first obtain the distribution of word frequency for each Chinese and English language book in our database. We find that the probability distribution *P*(*k*) of word frequency *k* for both Chinese and English books follows a power-law with scaling exponent *β* (Fig 1(a)),

$$P\left(k\right)∼k^{-\beta }.$$ (1)

**Fig 2:**
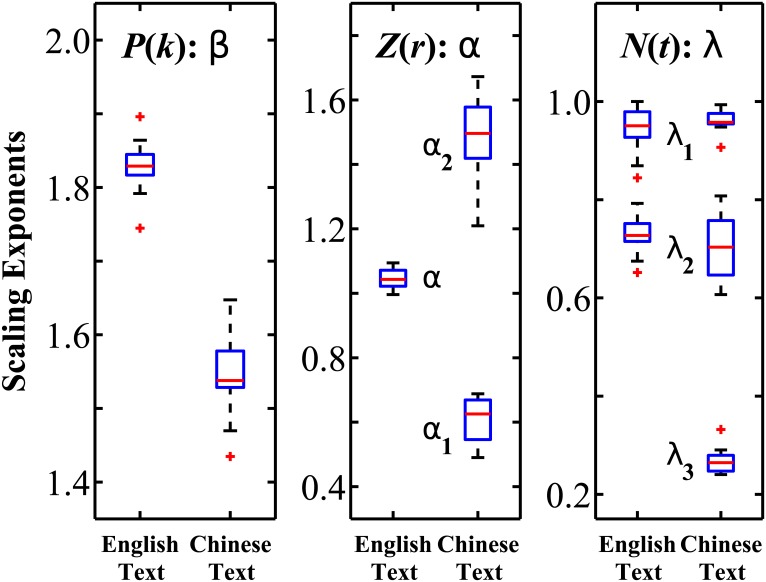
Statistical comparison of the scaling exponents derived from different scaling laws. Data show the group averaged values obtained for all books analyzed in Table 1. The central red mark in each box indicates the median value for the corresponding scaling exponents *β*, *α* and λ shown in three panels. Bottom and upper edges of each box denote the 25th and 75th percentiles correspondingly, while the whiskers extend to the most extreme data points not considered outliers; outliers are plotted individually by the symbol “+” in red color. The exponent *β* of the probability distribution *P*(*k*) is obtained in the range of word frequency *k* ∈ [1, 10^3^] for both Chinese and English texts. The Zipf’s law scaling exponent *α* for English books is obtained in the range of word frequency rank *r* ∈ [3, 2 × 10^3^], while for Chinese books *α*_1_ is obtained for small and intermediate scales *r* ∈ [3, 100] and *α*_2_ for large scales *r* ∈ [2 × 10^2^, 2 × 10^3^]. Heaps’ scaling law for both languages exhibits several scaling regimes with the exponent λ_1_ obtained for short scales of text length of *t* ∈ [1, 100] for both languages, λ_2_ in the scales of *t* ∈ [10^2^, 2 × 10^4^] for English texts and *t* ∈ [10^2^, 3 × 10^3^] for Chinese texts, and the exponent λ_3_ in long scales of *t* > 10^4^ for Chinese texts only. Our analyses indicate significant differences in all three scaling exponents *β*, *α* and λ between written texts in the Chinese and English language.

Our analyses show that Chinese language texts exhibit higher percentage of high frequency words compared to English texts. This is reflected by the significantly lower value of the scaling exponent *β* = 1.55 ± 0.06 (group mean ± standard deviation) for Chinese books, compared to *β* = 1.83 ± 0.04 for English books with Student’s *t*-test *p* < 0.01, showing statistically significant difference (Fig 2, fitting range *k* ∈ [1, 10^3^]).

We next perform Zipf’s rank analysis, and we find that words organization in both Chinese and English language texts obeys the Zipf’s law, i.e. the normalized word frequency *Z*(*r*) exhibits a power-law behavior as a function of the word frequency rank *r* characterized by a scaling exponent *α*,

$$Z\left(r\right)∼r^{-\alpha }.$$ (2)

Our analyses show that while English texts exhibit a power-law with a single exponent *α* = 1.05 ± 0.03 for the entire fitting range of words frequency rank *r* ∈ [3, 2 × 10^3^], the Chinese language texts are characterized by a clear crossover in the Zipf’s scaling from regime with *α*_1_ = 0.60 ± 0.07 for high and intermediate frequency ranks *r* ∈ [3, 100] to a second regime with scaling exponent *α*_2_ = 1.48 ± 0.14 for lower frequency ranks *r* ∈ [2 × 10^2^, 2 × 10^3^] shown in Fig 1(b) (with Student’s *t*-test *p* < 0.01, showing significant differences when comparing *α*_1_ and *α*_2_ of Chinese language texts, as well as when comparing the scaling exponents between the Chinese and English language, Fig 2). In Table 2, we list the top 20 most frequently used English words and Chinese characters and their frequencies.

**Table 2:** Top 20 most frequently used English words and Chinese characters and their frequencies. A Chinese character can have different functions in the structure of a sentence and carry different meanings depending on the context, as shown in brackets following each Chinese character in the table. The frequencies are calculated using pooled data of all books in our database.

Rank	English		Chinese	
*r*	Word	Frequency *Z*(*r*)	Character	Frequency *Z*(*r*)
1	the	0.0595	一(a, one, whole)	0.0174
2	of	0.0328	人(human, fellow)	0.0163
3	and	0.0304	了(end, finish, understand)	0.0155
4	to	0.0223	不(no, non-, without)	0.0154
5	a	0.0219	来(come, ever since)	0.0121
6	in	0.0187	说(say, persuade, theory)	0.0110
7	that	0.0130	道(path, say, doctrine, Tao)	0.0106
8	I	0.0115	是(is, correct, this, yes)	0.0106
9	it	0.0115	的(of, target)	0.0102
10	he	0.0107	有(have, exist)	0.0084
11	his	0.0096	他(he, other)	0.0083
12	was	0.0084	我(I)	0.0081
13	with	0.0081	在(at, in, be, exist)	0.0074
14	as	0.0072	你(you)	0.0071
15	for	0.0071	这(this, now)	0.0071
16	is	0.0070	去(go, leave, remove)	0.0071
17	on	0.0064	大(big, important, old)	0.0070
18	you	0.0061	个(a, individual, size)	0.0069
19	but	0.0058	上(up, good, superior, on)	0.0062
20	be	0.0057	见(see, meet with, opinion)	0.0061

We also find that words organization of both Chinese and English language texts obeys the Heaps’ law. Specifically, we find the number of distinct words *N*(*t*) grows as a power-law with increasing text length *t* for all Chinese and English books in our database (Fig 1(c)):

$$N\left(t\right)∼t^{\lambda }.$$ (3)

Our results show that at short scales *t* ∈ [1, 10^2^] both Chinese and English texts exhibit practically the same scaling behavior with exponent λ_1_ ≈ 1 (Fig 1(c)) with: λ_1_ = 0.96 ± 0.02 for Chinese language books and λ_1_ = 0.94 ± 0.05 for English (Student’s *t*-test indicates no statistical difference with *p* = 0.34). To estimate the range of this linear growth regime for each Chinese and English texts, we determine the scale *t*_1_ at which the scaling exponent λ reaches 0.95, and we find significant difference in the range of linear growth between the Chinese and English texts (*t*_1_ = 130.2 ± 43.2 for Chinese and *t*_1_ = 78.6 ± 16.2 for English, with *p* < 0.01).

Further, we find that the Heap’s scaling law exhibits a crossover at *t* ≈ 100 from a linear to a sub-linear scaling regime characterized by a scaling exponent λ_2_ ≈ 0.7 for the intermediate and large scales: group average λ_2_ = 0.70 ± 0.07 for Chinese texts in the range of *t* ∈ [100, 3 × 10^3^], and λ_2_ = 0.73 ± 0.04 for English in the full range of intermediate and large scales *t* ∈ [100, 2 × 10^4^] (Fig 1(c)). Student’s *t*-test *p* = 0.28 indicates no significant difference between Chinese and English language in this intermediate scaling regime.

Notably, our analyses show that all Chinese texts exhibit a second crossover at large scale *t* ≈ 4 × 10^3^, corresponding to *N*(*t*) ≈ 2 × 10^3^ with a transition to a saturation regime characterized by a distinct scaling exponent λ_3_ = 0.27 ± 0.03. This saturation regime is not observed in the English texts, but is typical for Chinese texts (Fig 1(c)) and results from the limited number of distinct Chinese characters (considered as words in our analyses) that are predominantly used in Chinese language texts.

### Stochastic feedback model of words organization and text growth mechanism in Chinese language

To understand the mechanism underlying words organization leads to the empirically observed scaling laws and study the difference between Chinese and English languages, we introduce a stochastic feedback model that accounts for the probability of word occurrence and growth of new word (that has not appeared in the text yet) with increasing text length.

To guide our model construction, we perform an additional statistical analysis on all books in our database to determine how the frequency of words in a new part of the text depends on the frequency of words in the previous part of the text. First, we divide a text into two equal parts (Part I and Part II), and calculate the frequency *k* for each distinct word in Part I, then we count *n*(*k*) the number of distinct words that appears *k* times in Part I. We next count the total number of times *N*(*k*) when all these *n*(*k*) words in Part I would also appear in Part II of the text. Finally, we calculate *ϕ*(*k*) the average number of occurrence in Part II of words that appeared *k* times each in Part I:

ϕ(k)=N(k)/n(k). (4)

**Fig 3:**
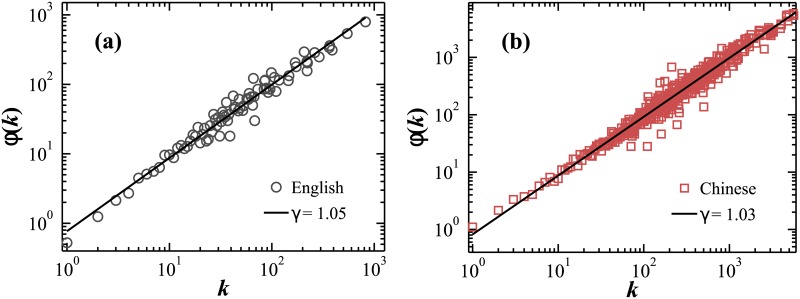
Empirical analyses of the word growth mechanism. Data show results for the first book of (a) English and (b) Chinese language listed in Table 1, revealing a scaling relation of the average number of occurrence *ϕ*(*k*) of a given word in the second half of a text, provided the frequency of occurrence of this word in the first half of the text is *k*. Both languages are characterized by a scaling exponent *γ* ≈ 1, indicating that words which appear with high frequency *k* in the first part of the text have also high-average occurrence in the rest of the text.

For both Chinese and English language books in Table 1, we find that *ϕ*(*k*) scales with *k* with an exponent *γ* ≈ 1 (Fig 3):

$$\phi \left(k\right)∼k^{\gamma }.$$ (5)

Hence, this analysis reveals a “rich-get-richer” mechanism in words growth, i.e., high frequency word in the “old” text tends to appears with similar high frequency in the “new” text. This behavior is consistently observed in all Chinese and English books in our database, and thus we introduce in our modeling approach this empirically derived functional relation for the organization of distinct words in written text.

To understand the origin of the observed scaling laws describing words structure in Chinese language texts, we simulate the process of language construction using the following stochastic feedback model of text growth. Given a text with a number of words, we grow the text following two different procedures:

**Procedure I**: with probability *p* we generate a new word that does not appear in the previous text and we add this word to the new text,
or
**Procedure II**: with probability 1 − *p* we select a word that is already present in the previous text and we add it to the new text.

Further, to model text growth dynamics based on the process of generating new distinct words that are not present in the prior text (Procedure I), we introduce a probability *p* that gradually changes with text length *t*:

$$p=k_{0}t^{-k_{t}},$$ (6)

where *t* is the growing text length. In our model simulation, for each book, we generate data length *t* = *T* that equals to the length of the corresponding empirical texts in Table 1.

At short text length *t*, the functional form of *p* in Eq (6) provides for a close to linear growth of the number of distinct new words. The values of the parameter *k*_0_ > 1 determine the range of the linear growth region where the text growth process includes only new words that are not present in the prior text. With text length *t* increasing, the probability *p* of adding distinctly new words to the text decreases, which is controlled by the scaling parameter *k*_*t*_ > 1 for Chinese texts in order to account for the saturation regime observed in the Heaps’ scaling law for very large scales of *t* shown in Fig 1(c) (for English texts the simulation parameter *k*_*t*_ ∈ (0, 1)).

In parallel to the text growth process that involves generating new distinct words with probability *p* (Eq 6), natural language text growth in our model involves also a second process of adding words that are already present in the prior text (Procedure II) with probability 1 − *p*. Guided by the empirical observations shown in Fig 3, in this procedure, our model incorporates a probability $\tilde{p}\left(i\right)$ to add a word *i* that is already in the text, where $\tilde{p}\left(i\right)$ has a positive dependence on the number of times *n*(*i*) that the word *i* already appears in the text:

$$\tilde{p}\left(i\right)=\frac{n{\left(i\right)}^{k_{p}}}{\sum_{i}n{\left(i\right)}^{k_{p}}}.$$ (7)

The values of the parameter *k*_*p*_ determine the degree of dependence between the probability of a word being selected and its number of occurrence in the already generated text.

**Fig 4:**
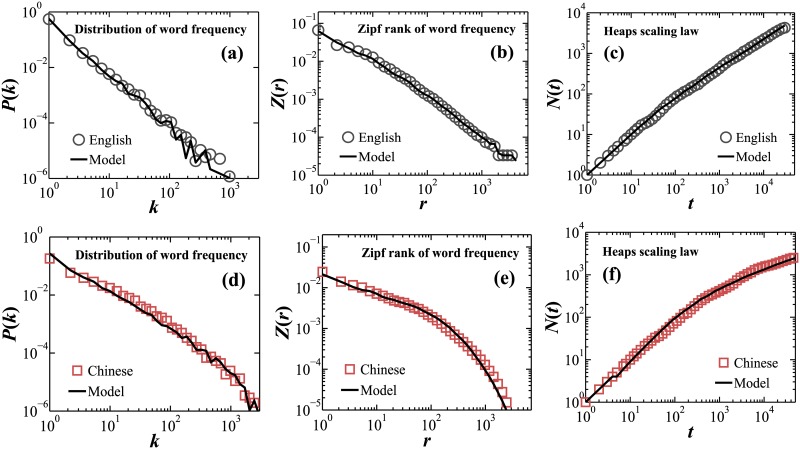
Empirical results and modeling simulation for Chinese and English language books. Scaling laws and model simulations for English book No. 1 (Table 1) are shown in panels (a), (b) and (c), and for Chinese book No. 1 (Table 1) are shown in panels (d), (e) and (f). Modeling parameters for all Chinese and English language books are given in Table 3.

**Fig 5:**
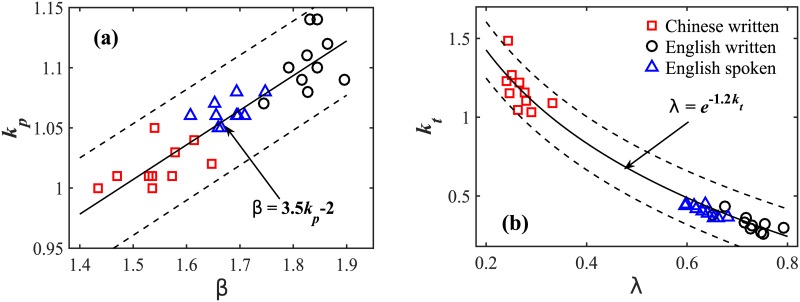
Functional relations between the empirically observed scaling exponents and model parameters. (a) Exponent *β* of the empirical probability distribution *P*(*k*) vs. model parameter *k*_*p*_ indicating linear functional dependence. (b) Heaps’ law scaling exponents λ_2_ for English books and spoken transcriptions, and λ_3_ for Chinese books vs. model parameter *k*_*t*_ indicating exponential functional dependence. Data points are obtained from the scaling analyses and simulation of all ten Chinese and English language books listed in Table 1, and English spoken language from Ref. [30]. The dotted lines indicate 95% confidence intervals of the data points obtained from empirical and model parameters for each separate book.

Fig 4 shows the scaling analyses results from our model simulation outputs, where model parameters were chosen to reproduce the empirical scaling properties we found for the first book in Table 1 in Chinese and English language. All fundamental scaling laws for *P*(*k*), *Z*(*r*) and *N*(*t*) with their specific scaling regimes are very well captured by our model. Moreover, our separate simulations for each book in the database reveal that:

1. There is a significant difference in the model parameter *k*_0_ values between the Chinese (*k*_0_ = 5.51 ± 1.56, group ave. ± std. dev.) and English language texts (*k*_0_ = 2.93 ± 1.01) (Table 3). This corresponds to our empirical findings that Chinese and English language have different linear growth regimes in *N*(*t*).
2. The model parameter *k*_*t*_ for Chinese texts has significantly higher values compared to English texts: *k*_*t*_ = 1.16 ± 0.17 for Chinese and *k*_*t*_ = 0.33 ± 0.05 for English, indicating a much lower growth rate of new distinct words with increasing text length in the Chinese language. Scatter plot diagram of the model parameter *k*_*t*_ vs. the empirical scaling exponent λ shown in Fig 5(b) for each Chinese and English book in our database reveals an exponential relationship between *k*_*t*_ and λ: $\lambda =e^{-1.2k_{t}}$.
3. Chinese language texts have significantly lower values of the model parameter *k*_*p*_ compared to English texts (*k*_*p*_ = 1.02 ± 0.02 for Chinese books and *k*_*p*_ = 1.10 ± 0.02 for English books, Table 3), indicating that Chinese texts exhibit weaker dependence between the probability $\tilde{p}\left(i\right)$ of a word *i* being selected and added to the text and its number of occurrence *n*(*i*) in prior text, which accounts for the scaling exponent *β* of the power-law probability distribution *P*(*k*). Scatter plot diagram of the model parameter *k*_*p*_ vs. the empirically derived scaling exponent *β* for each Chinese and English book in our database reveals a linear functional dependence between *k*_*p*_ and *β*: *β* = 3.5*k*_*p*_ − 2 (Fig 5(a)).

**Table 3:** Model parameters of Chinese and English books. The statistics show significant differences in the model parameters *k*_0_, *k*_*t*_ and *k*_*p*_ between Chinese and English texts, indicating differences in the dynamic process underlying the language structure, words organization and the occurrence of new words with text growth.

No.	*k*_0_		*k*_*t*_		*k*_*p*_	
	Chinese	English	Chinese	English	Chinese	English
01	4.68	2.34	1.10	0.30	1.01	1.14
02	4.05	2.16	1.05	0.26	1.03	1.09
03	5.51	2.76	1.22	0.30	1.00	1.10
04	4.60	2.85	1.09	0.32	1.04	1.12
05	5.50	3.28	1.16	0.36	1.02	1.09
06	5.95	3.11	1.27	0.38	1.01	1.08
07	4.16	2.79	1.03	0.33	1.00	1.10
08	5.17	2.54	1.15	0.31	1.01	1.11
09	5.93	1.94	1.23	0.27	1.05	1.14
10	9.52	5.54	1.48	0.44	1.01	1.07
group mean ± std. dev.	5.51 ± 1.56	2.93 ± 1.01	1.18 ± 0.13	0.33 ± 0.05	1.02 ± 0.02	1.10 ± 0.02
Student’s *t*-test	*p* < 0.01		*p* < 0.01		*p* < 0.01	

Note that, in Fig 5 we also include the results for English spoken language we analyzed in a previous study [30]. The data points obtained from English spoken language fall nicely on the fitting curves, which further validate the functional relation we establish in this study between the empirically observed scaling exponents and the model parameters.

## Conclusion

Our analyses of Chinese and English books indicate that both language forms obey power-law in the probability distribution of word frequency, as well as Zipf’s law and Heaps’ law, however with different scaling characteristics. Specifically, words organization in the Chinese language (i) is characterized by a significantly lower exponent for the probability distribution of words frequency, (ii) exhibits a pronounced crossover in the Zipf’s scaling law of words frequency rank, and (iii) obeys the Heaps’ scaling law exhibiting a similar growth rate in the number of new distinct words with increasing text length for short and intermediate scales compared to English texts, however with a unique for the Chinese language saturation regime when the number of distinct words (Chinese characters) reaches about 4000. The total number of Chinese vocabulary is approximately 90 thousand, and the number of commonly used Chinese characters is about 4500, which accounts for the Heaps’ law saturation regime at long text length. In contrast, the vocabulary size of the English language [31] is much larger than that of the Chinese language, thus the average text length of English books may not be sufficient to manifest a saturation regime as observed for Chinese language books. Our empirical findings and model simulations indicate a clear difference in the dynamics of language construction process between Chinese and English language forms, associated with higher concentration of high frequency words and lower occurrence of distinct new words in Chinese texts. The presented model successfully accounts for the different lexical generation mechanisms in Chinese and English. Our simulation results on words generation and text growth are in agreement with the empirical findings of three distinct scaling laws, and confirm that these laws accurately represent the complex dynamics of words organization in the Chinese and English language. The proposed here stochastic feedback model for text generation can be generally applied to other dynamic growth processes in complex systems characterized by scaling laws.
